# Fast Industrial Inspection of Optical Thin Film Using Optical Coherence Tomography

**DOI:** 10.3390/s16101598

**Published:** 2016-09-28

**Authors:** Muhammad Faizan Shirazi, Kibeom Park, Ruchire Eranga Wijesinghe, Hyosang Jeong, Sangyeob Han, Pilun Kim, Mansik Jeon, Jeehyun Kim

**Affiliations:** 1 School of Electronics Engineering, College of IT Engineering, Kyungpook National University, 80 Daehak-ro, Bukgu, Daegu 41566, Korea; faizanshirazi110@gmail.com (M.F.S.); pepl10@knu.ac.kr (K.P.); eranga@knu.ac.kr (R.E.W.); msjeon@knu.ac.kr (M.J.); 2 Oz-tec Co. Ltd., Office 901, IT Convergence Industrial Building, 47 Gyeongdae-ro, 17-gil, Bukgu, Daegu 41566, Korea; hsjeong@oz-tec.com (H.J.); syhan@oz-tec.com (S.H.); pukim@oz-tec.com (P.K.)

**Keywords:** OCT, LCD, optical thin film, industrial inspection, GPU

## Abstract

An application of spectral domain optical coherence tomography (SD-OCT) was demonstrated for a fast industrial inspection of an optical thin film panel. An optical thin film sample similar to a liquid crystal display (LCD) panel was examined. Two identical SD-OCT systems were utilized for parallel scanning of a complete sample in half time. Dual OCT inspection heads were utilized for transverse (fast) scanning, while a stable linear motorized translational stage was used for lateral (slow) scanning. The cross-sectional and volumetric images of an optical thin film sample were acquired to detect the defects in glass and other layers that are difficult to observe using visual inspection methods. The rapid inspection enabled by this setup led to the early detection of product defects on the manufacturing line, resulting in a significant improvement in the quality assurance of industrial products.

## 1. Introduction

The supply and demand of liquid crystal display (LCD) panels has increased in the last decade with the rapid development of display technology, along with the use of smart phones, notebooks, and LCD TVs. The large-scale production of LCDs, especially for smart phones and tablets with emerging display technologies, emphasized the need for fault detection at an early stage in the manufacturing process of electronic devices. The display production industry is expanding with an increase in facility investment. Therefore, fast, reliable and efficient LCD inspection equipment is essential for the current manufacturing industry. Reliable inspection equipment can detect and analyze defects and possible causes in the fabrication process, minimize production-line stoppages, and ensure customer satisfaction by increasing LCD reliability.

The measurement technology has high demands to maintain the quality assurance of the product. To obtain success, the metrology techniques are believed to be non-destructive and non-contact, along with automated image processing and analysis. Current LCD inspection techniques employ the visual inspection method, machine vision with a charge-coupled device (CCD) camera–based inspection, or electronic inspection after fabrication of signal lines or pixel electrodes [1,2,3,4,5,6]. From these techniques, the defect location corresponding to the top surface of a display panel can be detected, but the type and location of a defect in sub-layers cannot be identified [7,8]. Similarly, X-rays, computed tomography, and ultrasonic imaging are used in the material industry but none of them provides depth resolution at the micrometer scale [9,10]. Since an LCD is composed of several optical thin films, an inspection method that gives tomographic information for defect identification is required. Optical thin films are used in many applications, from the display industry to printed electronics.

Optical coherence tomography (OCT) is a promising non-invasive and non-destructive modality used for the imaging of microstructures with micrometer resolution. OCT was first reported by Fujimoto for an in vitro retinal study in 1991 [11]. It has been utilized for the early diagnosis of diseases in a non-destructive manner [12,13]. OCT has been widely used in different medical applications such as ophthalmology [14,15,16,17], dermatology [18], dentistry [19], otolaryngology [20,21,22], brain cancer studies [23], and in agricultural applications [24,25,26]. Several studies have demonstrated techniques to improve axial and transverse resolution without reducing the depth range of an OCT system [12,27,28,29].

With the development of novel OCT systems, the diversity of OCT applications has been expanded from the biological domain to industrial investigations. In industrial fields such as touch-screen panel inspection, verification of pearls, and identification of counterfeit notes [30,31,32], OCT has played an important role in the accurate identification of defects. Similarly, several reports have described OCT applications as inspection modalities in the industrial field [33,34,35,36,37,38]. In order to scan large areas on a sample, OCT requires much time to scan the whole sample by dividing it into segments. Therefore, there is a need to scan large areas in a reduced amount of time. A multi-beam setup for retinal imaging was reported which is capable of scanning a wide field of 10 mm in the back of the eye [39]. This setup uses multiple optical sources with a shared line scan sensor. By employing the same strategy for wider areas, large scanning is limited in terms of geometrical optics. Similarly, in the case of dual-beam OCT and space division multiplexing, the occurrence of cross-talk can be considered as the limiting factor. Additionally, due to the presence of two or more images on the same frame, high reflection at the defect position can affect the information of the other parts. As a result, the inline continuous inspection of large scanning area coverage is difficult to obtain due to these limitations. Considering these factors for large scanning, there is a need to develop an industrial inspection system with large imaging coverage for the automatic inline inspection of a whole sample in real time.

In this study, a parallel scanning method is used to scan a complete optical thin film panel similar to an LCD to reduce the inspection time. The large lateral range of the OCT system is utilized to scan the wide area on the sample instead of segment-wise scanning. Two identical spectral domain (SD)-OCT systems are employed in parallel to scan a sample. In industrial processing units, there is a strong requirement for fault detection at an early stage so that the quality assurance of the final product is maintained. OCT provides cross-sectional information with high accuracy and can detect faults in optical thin films that cannot be detected using visual inspection. Therefore, this study describes how OCT systems can be applied via parallel scanning to improve fault detection in the manufacturing process of display panels.

## 2. Materials and Methods

### 2.1. Hardware Setup

**Figure 1:**
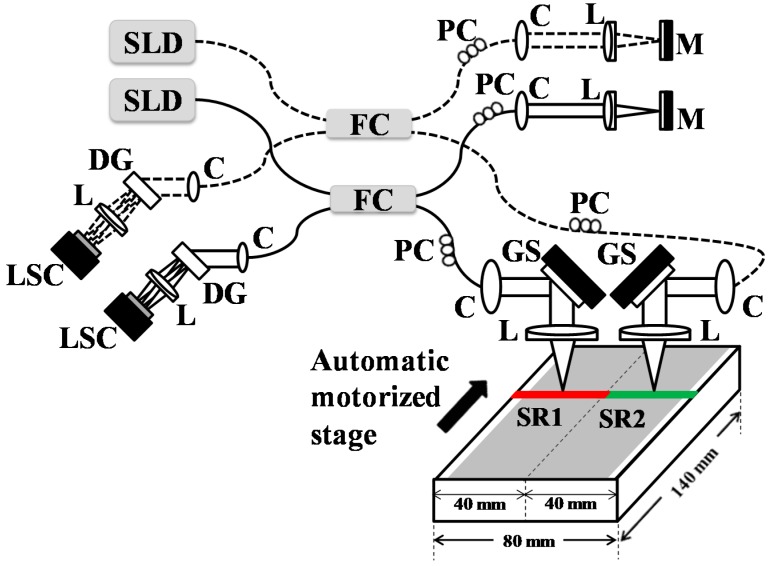
Optical architecture of two identical SD-OCT systems and sample stage. The red and green lines are respective scan ranges of two sample heads; Arrow indicates the direction of automatic motorized stage; C: collimator; DG: diffraction grating; FC: fiber coupler; GS: galvanometer scanner; L: achromatic lens; LSC: line scan camera; M: mirror; PC: polarization controller; SR1: scanning range 1; SR2: scanning range 2; SLD: super-luminescent diode.

Figure 1 provides a detailed schematic diagram of the proposed SD-OCT system for parallel scanning of a single sample. Two sample heads are connected to individual spectrometers through fiber couplers, and the SD-OCT systems that use fiber-based Michelson interferometers with infrared light sources and reference arms are shown in Figure 1. These two SD-OCT systems are considered as SD-OCT-1 and SD-OCT-2, with sample scanning head 1 and sample scanning head 2, respectively. The center wavelength of the source was 850 nm with 55 nm bandwidth, giving an axial resolution of 5 µm. Two super-luminescent diodes (EXS210068-01, Exalos Ltd., Schlieren, Switzerland) are connected to the two systems by fiber couplers. The detection part of each system contains a 12-bit complementary metal oxide line-scan camera (spL2048-140km, Basler, Ahrensburg, Germany) with 70,000 lines/s effective line rate and 2048 pixels, a transmission type diffraction grating (1800 lines/mm, Wasatch Photonics, Logan, UT, USA), achromatic lens (AC508-075-B, Thorlabs, Newton, NJ, USA), and a collimator. The optimized diffraction angle of grating is 46.05° for maximum diffraction efficiency. The fiber couplers split the light into respective sample and reference arms. For transverse scanning, a mirror with a focusing lens is used in each reference arm, while a 1-D (one dimensional) galvanometer mirror scanner (GVS001, Thorlabs, Newton, NJ, USA) with an objective lens is utilized in each sample head. The galvanometer mirror scanner is aligned at the back focal plane of the objective lens in each respective sample head. A frame grabber (PCIe-1433, National Instruments, Austin, TX, USA) is used to acquire the output from the line-scan cameras on a personal computer. The drive signals to the galvanometer mirror scanners and the trigger signals to synchronize the two cameras are generated by a personal computer through a data acquisition board (PCIe-6321, National Instruments, Austin, TX, USA). The two cameras are connected in base configuration to a single frame grabber. The sample scanning is carried out by two sample heads to acquire individual B-scan images. Each sample head used a scanning range of 41 mm, and the two sample heads together were able to scan 80 mm with a 1 mm overlapping distance. The illumination power over the sample surface is 2 mW, detection sensitivity is 105 dB, imaging range is 3 mm, axial resolution is 7 µm and transverse resolution along fast axis is 41 µm while along slow axis is 87.5 µm. In this configuration, two spectral domain optical coherence tomography (SD-OCT) systems are connected for simultaneous large-field scanning.

**Figure 2:**
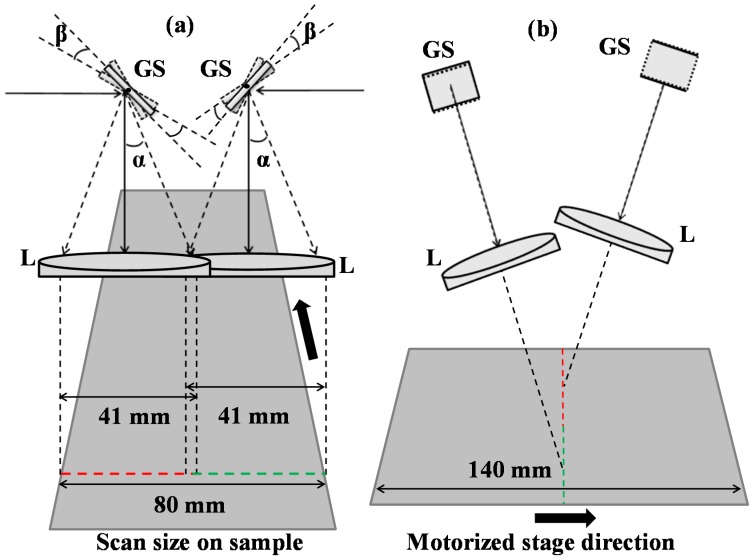
Schematic diagram of a two-sample-arm scanning head setup: (**a**) scan view in the x-direction; (**b**) scan view in the y-direction.

Figure 2 shows two sample-head scanning arrangements for industrial applications of the SD-OCT system for large-area scanning. Figure 2a,b elaborates the simultaneous scanning setup of two sample heads along x- and y-directions, respectively. Two identical SD-OCT systems are connected with their respective sample heads. Identical achromatic scanning lenses (AC508-100-B, Thorlabs, Newton, NJ, USA) of 100 mm focal length are used in the two samples as well as in the reference arms. The two sample heads in this setup were connected with rotational and translational stages to precisely align them. The two OCT scanning regions were aligned to form a large lateral scan by manually adjusting the sample heads through respective stages. The optical beam is illuminating on the sample surface at near normal angle as shown in Figure 2b to collect high reflection at different depths from sample. Due to the limitation on the geometric aspect and size of the scan heads, the optical beam illuminating the sample surface at near normal incident angle can cause discontinuity on the cross sectional OCT images. However, owing to the optical transparency and the low attenuation coefficient of the sample material, the penetration was high and the discontinuity on the cross-sectional OCT images in the deep regions was not much observed. With the help of IR card, the two sample heads are adjusted as much as possible to avoid any discontinuity in the combined image using feedback from OCT images. Galvanometer scanner-based optical inspection arms scan the sample in a transverse fast direction, while the automatic motorized stage moves the sample in a lateral slow direction. The speed of the motion and the galvanometer scanning frequency is controlled by a graphical user interface. The relation between the mechanical angle β and optical angle α is given as α = 2β. The galvanometer scanner has an analog input range of ±10 V with an input scale factor of 0.5 V per degree and maximum scan angle of ±20°. For an input voltage of ±3.15 V, the mechanical angle β is ±6.3°, while the optical angle α is ±12.6°. The scan range, corresponding to this angle with a 91.5 mm back focal length of lens, is approximately 41 mm. In this setup, the scanning of the two individual sample-heads overlapped by 1 mm. This overlapping gives some additional scan area common to both OCT images. Both sample-heads are tilted and adjusted to scan the sample without any information loss. A line rate of 42,000 A-scans/s is used for data acquisition through Basler line scan camera. A complete scanning of an optical thin film sample of dimension 80 mm × 140 mm takes approximately 38 s with a frame rate of 42 B-scans per second. Each individual sample head scanned its 41 mm × 140 mm area with 1000 A-scans × 1600 B-scans to make one C-scan (3D scan) of half of the sample panel area. For this 3D scanning, the linear motorized stage moved with a speed of 3.7 mm/s in a lateral slow scanning direction.

### 2.2. Software Setup

**Figure 3:**
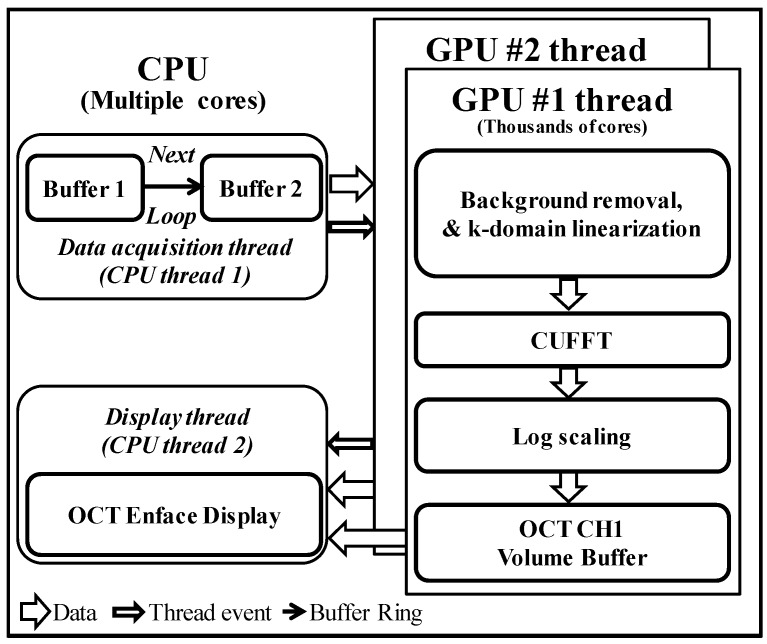
GPUs signal processing architecture of the proposed industrial SD-OCT system for optical thin film inspection.

The data acquisition, process, and display program is written in LabVIEW 2013 for this wide-field scanning system. A compute unified device architecture (CUDA) with two graphics processing units (GPU) is used for fast data processing and display of OCT images. Figure 3 shows the programming architecture of the SD-OCT systems that includes the data flow between the CPU and the GPU, events thread, and processing. The data acquisition-thread first stores the incoming two-dimensional raw signals into buffer 1 allocated in random access memory in the CPU. After that, data is copied into buffer 2, which continuously transfers data to the GPU. The second buffer is used to avoid any temporal delay in data acquisition events. The signal processing thread copies the frame data stored in buffer 2 of the CPU memory through the PCI express 2.0 × 16 bus interface into the GPU memory. The signal processing is divided into CUDA sub-processors to process the signal for OCT. Data processing includes background noise removal, k-domain linearization, and fast Fourier transform. Background noise is removed by subtracting the acquired signal from the reference signal. Full-range k-domain linearization is used to remove the non-linearity in the raw signal [40,41]. The full-range k-domain linearization is employed in both spectrometers to remove the nonlinearity in axial direction. The filtered spectrum with a narrow linewidth (~0.5 nm) is detected to the line-scan camera. The wavelength scanning filter selects narrow linewidth light by translating the slit through broadband spectrum. At each step of slit translation, the central wavelength of the filtered light is obtained from optical spectrum analyzer (OSA) along with respective pixel position from spectrometer, automatically. By utilizing the wavelength and pixel position, the k-linear pattern is generated. A lookup table is generated to linearize the measured spectrum in the k-domain. The lookup table covers the full pixel range. As a result, no additional fitting algorithm is required during image acquisition and display. Since both spectrometers were individually linearized in such a way to match the features in axial direction by generating lookup tables. Therefore, the images obtained by each probe head had almost identical features. Additionally, the identical optical components with almost identical fiber lengths in sample and reference arms are used to avoid dispersion which can also affect the axial resolution in conventional fast Fourier transformed data [42]. After log scaling, the processed data is sent back to the display thread which displays the reconstructed OCT B-scan image in real time. This process is continued to increase the data acquisition speed with a high B-scan frame rate. GPU-based fast image processing is important issue in the real-time fault inspection environment. The display thread is used to display the OCT cross-sectional as well as enface images of the sample under inspection. This thread also stores the display data for post-processing.

**Figure 4:**
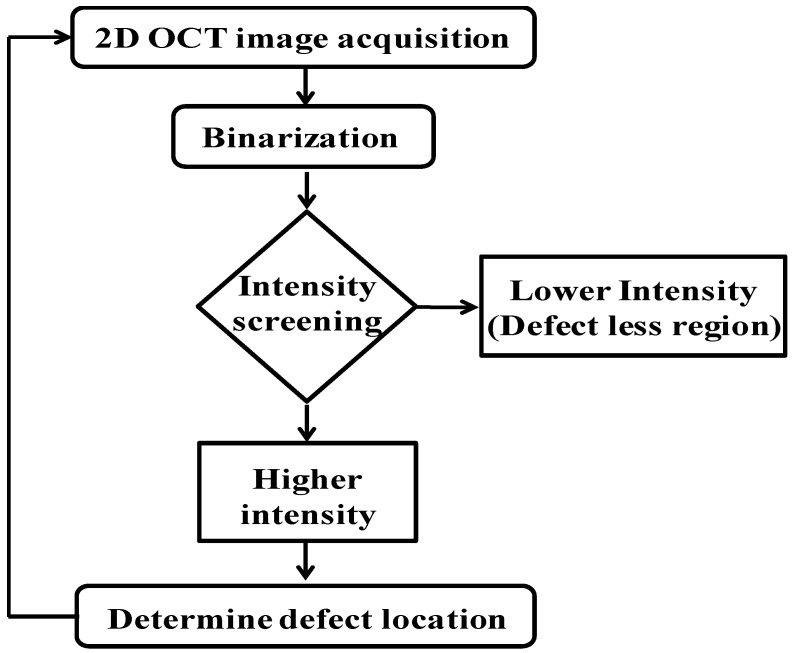
Flowchart of the algorithm for automatic defect detection.

A software-based defect detection algorithm was developed for the inspection of the abnormal defects. The algorithm is capable of analyzing the particular defective region, defect dimensions, and the number of defects in each sample. A flow chart including the processing steps is shown in Figure 4 to gain a better understanding of the inspection algorithm. Initially, a standard OCT image is acquired and was binarized to classify the intensity and the magnitude of the defect. A pre-determined intensity value is utilized as the intensity level to classify the defectiveness. The total intensity, which exceeds the aforementioned threshold value of the binary image, can be characterized as a defective region, and in this regard, the corresponding defective location can be screened with a high intensity. The least intensity characterizes the non-defective location in the binary image. As the first step in the inspection procedure, the tracking method expands in horizontal and vertical directions to confirm the boundary of the defective region. Thus, the boundaries of the defective and non-defective regions were determined according to various intensity levels and this enabled the algorithm to confirm the precise location of the defective region rigorously. If the detection method meets with a higher intensity position, the corresponding intensities as well as the magnitude of the area are confirmed as an abnormal region of the detection plane. Once the inspection of a one sample is concluded, the algorithm is repeated and continuously repeats for the further samples. This algorithm at the same time provides the quantitative detail about the defect location in each B-scan, number of defects, and size of defect. After complete scanning of sample, the cross-sectional as well as enface information of defect location can be traced precisely. The signal to noise ratio is maintained to minimize the detection of noise as defect. In the data sets present, 1600 B-scan images are acquired from each SD-OCT system with the dimension of 1000 A-lines in each B-scan image. The total number of defects detected in whole scanning was 20 times with some B-scans contain multiple defects. A log file is generated to locate the defect position along with B-scan number. All the OCT images from individual scanning heads along with merged OCT images are stored in the system memory. After inspection, a user can analyze the type, size and location of defection by scrolling on the saved images. Since the axial and transverse resolution of this large scan system is 7 μm and 41 μm, respectively, therefore the smallest defect that system can reliably detect is equal to the resolution of the system. In this experiment the defect size ranges from 10 μm to 2 mm.

### 2.3. Materials

**Figure 5:**
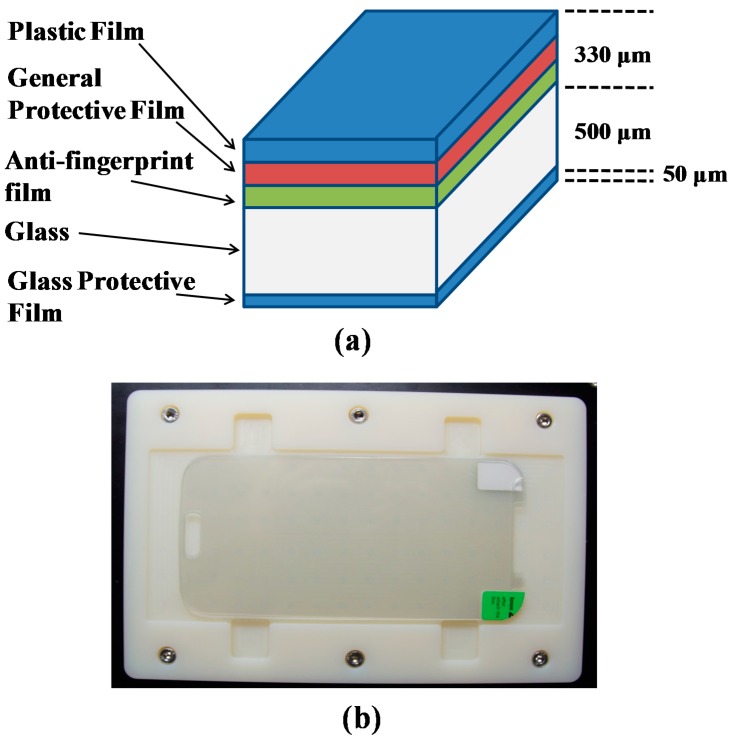
Optical thin film panel structure with camera image: (**a**) optical thin film panel with individual layer thickness; (**b**) optical thin film panel placed on the scanning stage.

An optical thin film sample panel is designed to test the feasibility of the system for real-time inspection. Figure 5a shows the layer thickness and different layers of the sample. The top layer is composed of general protective film, plastic film, and anti-fingerprint film. The second layer is composed of glass, and the last layer is glass protective film for glass protection. The thicknesses of these layers are different according to the specification of the sample panel. In this study the sample has corresponding thicknesses as shown in Figure 5a. The first layer consists of three sublayers with a total thickness of 330 μm. The middle layer is composed of glass with 500 μm thickness. The bottom layer of glass protection is 50 μm thick. In OCT, cross-sectional information is obtained owing to a change in the refractive index. If the layer is thick and composed of a single refractive index, then only the layer border is visible in OCT images. Similarly, if there is a defect in that layer, that defect is observable owing to a change in the refractive index. Figure 5b shows a representative sample image placed on the motorized scanning stage. The physical dimensions of the sample are 70 mm × 140 mm.

### 2.4. Graphical User Interface

**Figure 6:**
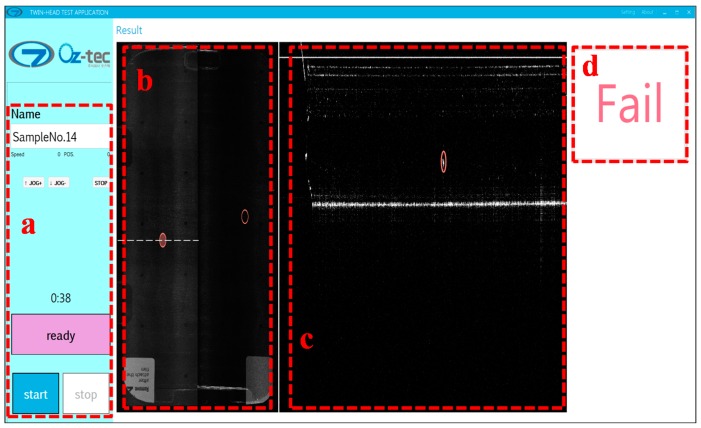
Optical thin film panel enface image with real-time fault detection: (**a**) system setup containing user control buttons and indications; (**b**) enface image of sample obtained from two scanning heads; (**c**) cross-sectional OCT image with fault detection in panel along white dashed line in (**b**); and (**d**) “Pass” or “Fail” indication based on fault detection.

Figure 6 shows the graphical user interface used for the real-time fault inspection of the sample. The optical thin film containing multiple layers as described in Figure 5 is investigated using our proposed system. The graphical user interface (GUI) developed by Oz-tec Co., Ltd. is used for the real-time fast inspection of the sample. Figure 6a shows the control parameters selected to operate the system, while Figure 6b,c shows the OCT enface and cross-sectional images of the sample, respectively. The image shown in Figure 6c is the cross-sectional image along the white dashed line in Figure 6b. The enface image is obtained after scanning the complete sample using two sample heads. The red-colored encircled positions indicate the detection of a defect in the sample. If a defect is detected in the sample, then an indication of “Fail” appears at the (d) position on the GUI; otherwise, a “Pass” indication will display if no defects are found. All defect positions can be seen on the enface image simultaneously, while the nature and position of a defect in different layers could be identified by viewing the cross-sectional OCT image.

## 3. Results and Discussion

**Figure 7:**
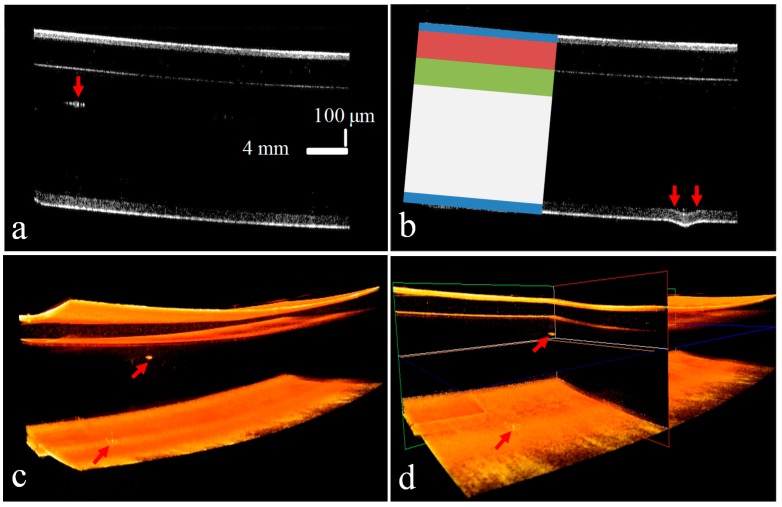
Results of sample head-1 of the SD-OCT-1 system with defects are indicated with red arrows: (**a**) cross-section view through sample head-1 where the defect in the glass layer can be seen clearly; (**b**) defect in the bottom layer; (**c**) 3D scan result of the defect; and (**d**) ortho-sliced view of defect identified in (**a**).

Figure 7 shows the experimental results of the sample using sample head-1 in the SD-OCT-1 system where defects are detected. The optical thin film contains multiple layers of varying thickness as shown in the figure. The purpose of the top three layers of the optical thin film panel is to protect against defects being caused by external interactions. The most important layer of the panel is composed of glass 500 μm thick, and again, on the bottom, a protective film is used to prevent the glass from damage. Figure 7a shows the cross-sectional image of an identified defect in the second layer, while Figure 7b shows a bending defect at the bottom layer as indicated by the arrows. Figure 7c shows the 3D structure of a defect in the optical thin film. Figure 7d shows the ortho-sliced view of the panel. The detailed architecture of the optical thin film is shown with color-coded layers in Figure 7b. The blue color indicates the top and bottom plastic film layer, the red color shows the general protective film layer, the green color is anti-fingerprint film, while the white color represents the glass layer. The arrow at the bottom layer in the 3D structure indicates the bending defect as shown in Figure 7b. The SD-OCT system provides cross-sectional information on panels with micrometer resolution. This information can be used to enable the automatic selection of a defective sample and to discard the sample at an early stage of the manufacturing process. The cross-sectional hidden defects in the glass layer, which could not be identified by visual inspection or any other technique, are clearly detected.

**Figure 8:**
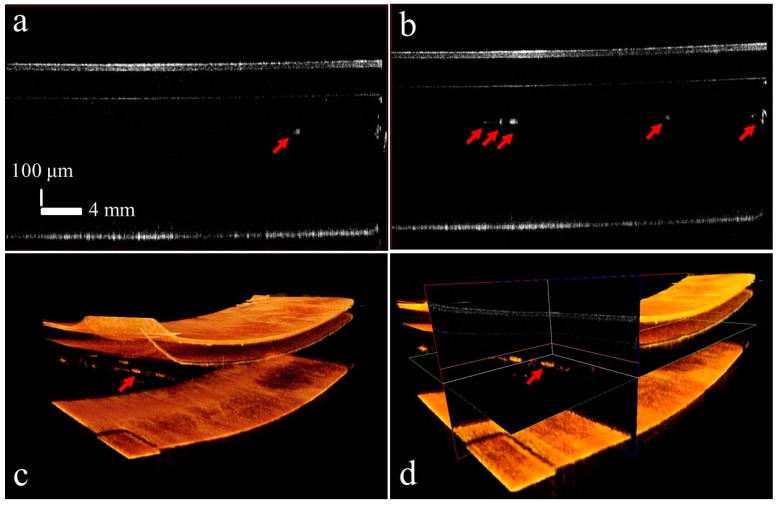
Results of sample head-2 of the SD-OCT-2 system with defects identified with red arrows: (**a**) cross-sectional view of single-point defect in the glass layer; (**b**) multiple defects in the glass layer; (**c**) 3D scan result showing multiple defects in the glass layer; (**d**) ortho-sliced view of defect.

Similarly, Figure 8 shows the experimental results using sample head-2 with SD-OCT-2. Figure 8a,b shows the cross-sectional images of an optical thin film panel with single and multiple defect identification in the glass layer, respectively. Figure 8c provides a 3D view of a sample with multiple defects in the middle glass layer; a slight tilt at the front corner of the sample can also be observed. Figure 8d shows the ortho-sliced view of the sample. Figure 7 and Figure 8 demonstrate the overall success of the SD-OCT systems with parallel scanning to detect cross-sectional hidden defects in the glass layers. This setup of parallel scanning of a wide area can play an important role in the panel fabrication industry in the rapid identification of defective optical thin film and LCD products.

**Figure 9:**
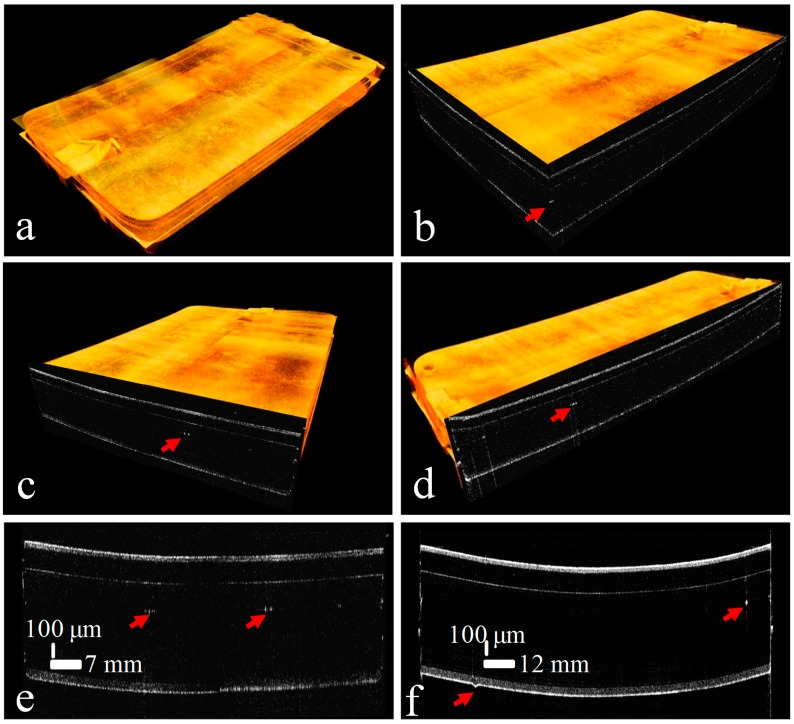
Merged volumetric images from two SD-OCT systems with defects identified with red arrows: (**a**) volumetric image of complete thin film panel scanned by two scanning heads simultaneously; (**b**) the ortho-sliced view of the volumetric image; (**c**) the ortho-sliced cross-sectional view along the transverse fast-scanning direction of two sample heads; (**d**) the ortho-sliced cross-sectional view along the motorized lateral slow-scanning direction; (**e**) merged cross-sectional image by two sample heads; and (**f**) cross-sectional image along lateral slow-scanning direction.

Figure 9 shows the merged volumetric OCT images acquired from two SD-OCT systems with ortho-sliced and cross-sectional images. At the corner and middle edge of the panel, scotch tape is used to stabilize the panel on a linear motorized stage. The defective positions in the optical thin film panel are indicated with the arrows. The defects in the middle layer and bottom layer can be analyzed easily.

## 4. Conclusions

The industrial setup for a parallel scanning scheme was demonstrated in this paper. In order to maintain the frame rate and lateral resolution, two SD-OCT systems were utilized to scan large areas in parallel. Transverse fast scanning of samples was performed with a galvanometer scanner, while a motorized stage was used for lateral slow scans. Optical thin film samples were investigated with the proposed system and defects were identified. The defects in the glass layers and layer damage were also observed. The incorporation of two SD-OCT systems reduced typical scan times by 50% as compare to a single SD-OCT system [38,43]. This setup can be utilized in the display electronics industry for fast error detection at early stages of the manufacturing process and for the resultant rapid removal of defective products in the fabrication of optical films, display panels, glass panels, and other transparent materials. As a result, one can gain more direct and relevant information as compared to other inspection techniques. This setup can be helpful for fast, reliable, and efficient defect identification in the LCD panel production line. As the quality assurance of the production line can be increased dramatically. In the future, we will use LCD samples to verify the performance of our system to detect stains, nicks, and bubble defects in multiple layers. Furthermore, after further improvements in the performance of our system, we will broaden the range of our investigations to enable this technology to be used for inline defect detection and identification of causal factors in products such as solar panels, flexible display panels, and other thin film objects.
